# Putative biomarkers for early diagnosis and prognosis of congenital ocular toxoplasmosis

**DOI:** 10.1038/s41598-020-73265-z

**Published:** 2020-10-07

**Authors:** Thádia Evelyn de Araújo, Luara Isabela dos Santos, Angelica Oliveira Gomes, Ana Carolina Aguiar Vasconcelos Carneiro, Anderson Silva Machado, Jordana Grazziela Coelho-dos-Reis, Vanessa Peruhype-Magalhães, Samantha Ribeiro Béla, Gláucia Manzan Queiroz Andrade, Daniel Vitor Vasconcelos-Santos, José Nélio Januário, Andréa Teixeira-Carvalho, Ricardo Wagner Almeida Vitor, Lis Ribeiro do Valle Antonelli, Eloisa Amália Vieira Ferro, Olindo Assis Martins-Filho, the UFMG Congenital Toxoplasmosis Brazilian Group UFMG-CTBG, beside the authors, Danuza O. Machado Azevedo, Ericka V. Machado Carellos, Luciana Macedo Resende, Roberta M. Castro Romanelli

**Affiliations:** 1 Instituto de Ciências Biomédicas, Universidade Federal de Uberlândia, Avenida João Naves de Ávila 2121, Santa Mônica, Uberlândia, MG 38408-100 Brazil; 2 Instituto René Rachou, Fundação Oswaldo Cruz, Avenida Augusto de Lima, 1715, Barro Preto, Belo Horizonte, MG 30190-002 Brazil; 3 Faculdade de Ciências Médicas de Minas Gerais, Alameda Ezequiel Dias, 275, Centro, Belo Horizonte, MG 27530130-110 Brazil; 4 Instituto de Ciências Biológicas e Naturais, Universidade Federal do Triângulo Mineiro, Rua Frei Paulino, 30, Nossa Sra. da Abadia, 38025-180 Uberaba, MG Brazil; 5 Departamento de Parasitologia, Universidade Federal de Minas Gerais, Avenida Presidente Antônio Carlos, Pampulha, Belo Horizonte, MG 662731270-901 Brazil; 6 Departamento de Microbiologia, Instituto de Ciências Biológicas, Universidade Federal de Minas Gerais, Avenida Antônio Carlos, 6627, Pampulha, Belo Horizonte, MG 31270-901 Brazil; 7 Departamento de Pediatria, Universidade Federal de Minas Gerais, Avenida Professor Alfredo Balena 190, Santa Efigênia, Belo Horizonte, MG 30130-100 Brazil; 8 Núcleo de Ações e Pesquisa em Apoio Diagnóstico (NUPAD), Universidade Federal de Minas Gerais, Avenida Professor Alfredo Balena 190, Santa Efigênia, Belo Horizonte, MG 30130-100 Brazil; 9 Departamento de Oftalmologia e Otorrinolaringologia, Faculdade de Medicina da UFMG, Belo Horizonte, MG Brazil; 10 Uveitis Unit, Hospital Sao Geraldo, Hospital das Clínicas, UFMG, Belo Horizonte, Brazil; 11 Department of Phonoaudiology, School of Medicine, UFMG, Belo Horizonte, Brazil

**Keywords:** Diagnostic markers, Prognostic markers, Cytokines, Immunology, Infectious diseases, Parasitic infection

## Abstract

In the present study we have evaluated the performance of several immunological biomarkers for early diagnosis and prognosis of congenital toxoplasmosis. Our results showed that ex vivo serum levels of CXCL9, and the frequencies of circulating CD4^+^CD25^+^ T-cells and *T. gondii*-specific IFN-γ^+^CD4^+^ T-cells measured 30–45 days after birth presented high accuracy to distinguish *T. gondii*-infected infants from healthy age-matched controls (Global Accuracy/AUC = 0.9; 0.9 and 0.8, respectively). Of note was the enhanced performance (Accuracy = 96%) achieved by using a combined stepwise analysis of CD4^+^CD25^+^ T-cells and CXCL9. In addition, high global accuracy (AUC = 0.9) with elevated sensitivity (Se = 98%) was also reached by using the total frequency of in vitro IFN-γ-producing *T. gondii*-specific T-cells (∑ IFN-γ^+^ CD4^+^ & CD8^+^) as a biomarker of congenital toxoplasmosis. Furthermore, the analysis of in vitro* T. gondii*-specific IL5^+^CD4^+^ T-cells and IFN-γ^+^NK-cells displayed a high accuracy for early prognosis of ocular lesion in infant with congenital toxoplasmosis (Global Accuracy/AUC = 0.8 and 0.9, respectively). Together, these findings support the relevance of employing the elements of the cell-mediated immune response as biomarkers with potential to endorse early diagnosis and prognosis of congenital ocular toxoplasmosis to contribute for a precise clinical management and effective therapeutic intervention.

## Introduction

The *Toxoplasma gondii* infection has a worldwide distribution, affecting about 25–30% of the human population^1–3^ . The seroprevalence may range from less than 10% to over 90%, depending on the nutrition and hygiene habits of populations from distinct geographical areas^1,4^. *T. gondii* infection may result from the ingestion or handling of undercooked/raw meat containing cysts or water and food contaminated with oocysts. Moreover, congenital toxoplasmosis may occur as consequence of primary infection during pregnancy, possibly resulting in fetal death and abortion or causing severe syndromes that include neurologic damage, neurocognitive deficits or chorioretinitis.

Despite the severity of the lesions that congenital toxoplasmosis can trigger, they are not always detectable during prenatal care. Thus, fetal damage caused by toxoplasmosis is detectable by ultrasound screening only when severe neurological anomalies are present. However, toxoplasmic chorioretinitis is not accessible by ultrasonography. The clinical manifestations of congenital toxoplasmosis may be absent in newborns, but long-term sequelae can be observed, including retinochoroiditis and neurological abnormalities. Moreover, no signs described in newborns with congenital disease are pathognomonic for toxoplasmosis^1^.

The diagnosis of congenital toxoplasmosis is generally established based on the use of several laboratorial methods, including the isolation of *T. gondii* from blood or body fluids, detection of parasite DNA and serological tests for detection of *T. gondii*-specific immunoglobulins. Although the detection of *T. gondii*-specific IgM and IgA, which do not cross the placenta, are considered good markers of congenital infection, these biomarkers show low sensitivity and cannot detect more than 75% of infected babies^1,5^. As *T. gondii*-specific IgG crosses the placenta and the clearance of maternally transferred IgG may take 6–12 months^1,6^, IgG is not a feasible laboratorial marker for congenital toxoplasmosis. In this sense, it is relevant to propose complementary diagnostic approaches to contribute for a precise clinical management and effective establishment of therapeutic intervention immediately after birth.

It is well known that besides eliciting a robust antibody response, congenital *T. gondii* infection results in long-lasting cell-mediated immune response, which involve a wide range of cell subsets and soluble molecules^5,7–14^. Surprisingly, the studies that investigated the potential role of cell immunity in diagnosis of congenital toxoplasmosis or methods with prognostic potential to predict the retinochoroidal lesion status are still scarce. It has been proposed that IgM screening at birth, followed by flow cytometric IgG avidity analysis at 30–45 days after birth, displays high performance for early serological diagnosis of congenital toxoplasmosis^15^. Moreover, the use of flow cytometric serology has been recognized as a potential method for early prognosis of ocular lesions in *T. gondii*-infected infants^16^.

It has been reported that high CD25 expression is detectable in almost all *T. gondii*-infected patients, including newborns^8^. Moreover, the analysis of other T-cell activation markers, such as HLA-DR and IFN-γ producing T-cells, has shown to be a useful approach for early and accurate diagnosis of congenital toxoplasmosis^9^. Aiming at improving the laboratorial diagnosis of toxoplasmosis, complementary methods using whole blood samples have been proposed. In this line, a simple test based on whole blood IFN-γ-release assay to assess the T-cell-mediated immunity in toxoplasmosis with high sensitivity and specificity have been described^5,11,17^.

In the present study, we evaluated a broad range of immunological mediators to be employed as biomarkers for early diagnosis and prognosis of the development of ocular lesions in infants with congenital toxoplasmosis. Using small volumes of whole blood samples, the performance of serum soluble mediators, ex vivo phenotypes of circulating leukocytes and intracellular cytokine profiles upon short-term in vitro stimuli were evaluated as single or combined stepwise biomarker platforms. As reported here, we found that the levels of CXCL9, and the frequencies of circulating CD4^+^CD25^+^ T-cells and *T. gondii*-specific IFN-γ^+^CD4^+^ T-cells measured 30–45 days after birth presented high accuracy to distinguish *T. gondii*-infected infants. Moreover, the combined stepwise analyses of CD4^+^CD25^+^ T-cells and CXCL9 or the total frequency of in vitro IFN-γ-producing *T. gondii*-specific T-cells further improve the accuracy of the diagnosis of congenital toxoplasmosis. Finally, the contemporaneous analysis of *T. gondii*-specific IL-5^+^CD4^+^ T-cells and IFN-γ^+^NK-cells serve as an early prognosis tool of ocular involvement in infant with congenital toxoplasmosis.

## Results

### Screening of serum chemokines and cytokines as complementary biomarkers for early diagnosis and prognosis of congenital toxoplasmosis

The performance of serum chemokines and cytokines as a complementary biomarker for early diagnosis of congenital *T. gondii* infection was first evaluated assessing the sensitivity (Se) and specificity (Sp) to segregate *T. gondii*-infected infants (TOXO) from healthy controls (CTL). Additionally, the ability of these serum biomarkers to provide an early prognosis assessment were also evaluated by comparing TOXO with retinochoroidal lesion (L) vs. TOXO without lesions (NL) as well as TOXO with active lesion (AL) vs. TOXO with cicatricial lesion (CL). The performance indices (AUC, Se and Sp) are presented in the Table 1, from higher to lower global accuracy (AUC). The specific cut-offs used for each biomarker were calculated by the ROC curve and are provided in the Table 1. Data analysis demonstrated that CXCL9 and CXCL10 display high accuracy (AUC > 0.8) to distinguish TOXO from CTL individuals. Low accuracy (AUC < 0.7) was found for serum chemokines and cytokines for early prognosis assessment by comparing NL vs. L or AL vs. CL (Table 1).

**Table 1:** Screening of serum chemokines and cytokines as complementary biomarkers for early diagnosis and prognosis of congenital toxoplasmosis.

Diagnosis				Early prognosis							
Biomarkers^a^ (pg/mL)	CTL × TOXO			Biomarkers^b^ (pg/mL)	TOXO (NL × L)			Biomarkers^c^ (pg/mL)	TOXO (AL × CL)		
	AUC	Se (%)	Sp (%)		AUC	Se (%)	Sp (%)		AUC	Se (%)	Sp (%)
**CXCL9**	**0.9**	**84**	**81**	IL-5	0.6	92	28	IL-12p70	0.6	91	30
**CXCL10**	**0.8**	**82**	**69**	TNF	0.6	78	50	IL-6	0.6	87	30
CCL5	0.6	83	42	CXCL8	0.6	68	50	CCL2	0.6	84	37
CXCL8	0.6	68	50	IL-6	0.6	62	61	CCL5	0.6	78	37
IFN-γ	0.6	58	65	CCL2	0.6	61	72	IL-17A	0.6	72	46
IL-12p70	0.6	56	61	IFN-γ	0.6	55	72	IL-4	0.6	63	52
IL-1β	0.6	52	65	CCL5	0.6	40	78	IL-1β	0.5	96	15
TNF	0.6	31	83	IL-17A	0.5	83	33	IL-10	0.5	94	21
IL-10	0.5	91	22	IL-1β	0.5	81	39	CXCL8	0.5	93	17
CCL2	0.5	91	23	IL-4	0.5	71	50	IL-5	0.5	93	27
IL-17A	0.5	91	22	CXCL9	0.5	68	50	IFN-γ	0.5	59	52
IL-4	0.5	85	30	IL-12p70	0.5	51	61	TNF	0.5	48	70
IL-5	0.5	51	70	IL-10	0.5	46	72	CXCL9	0.5	40	74
IL-6	0.5	42	74	CXCL10	0.5	13	100	CXCL10	0.5	31	86

CTL = Uninfected infant controls (n = 26); TOXO = Infants with congenital toxoplasmosis (n = 108); NL = no retinochoroidal lesion (n = 18); L = retinochoroidal lesion (n = 90); AL = active retinochoroidal lesion (n = 35); CL = cicatricial retinochoroidal lesion (n = 55); AUC = Area under the ROC curve; Se = Sensitivity; Sp = Specificity. Bold values and letters correspond to biomarkers with high accuracy (>0.8).

^a^Cut-off: CXCL9 = 6754; CXCL10 = 12,050; CCL5 = 937; CXCL8 = 3.3; IFN-γ = 1,565; IL-12p70 = 72; IL-1β = 197; TNF = 64; IL-10 = 98; CCL2 = 230; IL-17A = 795; IL-4 = 704; IL-5 = 451 and IL-6 = 1,861.

^b^Cut-off: IL-5 = detectable levels; TNF = 346; CXCL8 = 3.3; IL-6 = 986; CCL2 = 59; IFN-γ = 2,031; CCL5 = 1,295; IL-17A = 335; IL-1β = 83; IL-4 = 342; CXCL9 = 10,135; IL-12p70 = 275; IL-10 = 887 and CXCL10 = 8523.

^c^Cut-off: IL-12p70 = 4617; IL-6 = 3,460; CCL2 = 141; CCL5 = 2,215; IL-17A = detectable levels; IL-4 = 249; IL-1β = 1,065; IL-10 = 138; CXCL8 = 1.2; IL-5 = 2,914; IFN-γ = 1,414; TNF = 91; CXCL = 15,256 and CXCL10 = 24,694.

### Screening of circulating leukocyte subsets as complementary biomarkers for early diagnosis and prognosis of congenital toxoplasmosis

The absolute counts of white blood cells performed by automated hematological and the frequency of circulating monocytes, neutrophils, eosinophils, T-cells and B-cell subsets generated from ex vivo flow cytometry analysis, assessed 30–45 days after birth, were used to evaluate the sensitivity (Se) and specificity (Sp) to segregate *T. gondii*-infected infants (TOXO) from healthy controls (CTL). Moreover, the ability of these biomarkers to distinguish “NL from L” as well as “AL from CL” was also evaluated. The resultant indices (AUC, Se and Sp) are provided in the Table 2. Data analysis showed that frequencies of CD4^+^CD25^+^ T-cells, γδ T-cells, CD8^+^ T-cells, CD4^+^HLA-DR^+^ T-cells, CD8^+^HLA-DR^+^ T-cells, CD4^+^ T-cells discriminate TOXO from CTL individuals with high accuracy (AUC > 0.8). Moderate (AUC = 0.7) or low (AUC < 0.7) accuracies were found for comparisons between “NL vs. L” or “AL vs. CL” (Table 2).

**Table 2:** Screening of ex vivo circulating leukocyte subsets as complementary biomarkers for early diagnosis and prognosis of congenital toxoplasmosis.

Diagnosis				Early prognosis							
Biomarkers^a^ (% or MFI, counts)	CTL × TOXO			Biomarkers^b^ (% or MFI, counts)	TOXO (NL × L)			Biomarkers^c^ (% or MFI, counts)	TOXO (AL × CL)		
	AUC	Se (%)	Sp (%)		AUC	Se (%)	Sp (%)		AUC	Se (%)	Sp (%)
**CD4**^**+**^**CD25**^**+**^	**0.9**	**82**	**91**	CD3^−^CD16^+^ & CD3^−^CD56^+^	0.7	63	73	CD3^−^CD16^+^ & CD3^−^CD56^+^	0.7	82	62
**TCRγδ**	**0.8**	**82**	**76**	CD3^+^CD56^+^	0.7	59	80	CD3^−^CD16^+^CD56^+^	0.7	55	92
**CD8**^**+**^	**0.8**	**78**	**68**	CD3^−^CD16^+^CD56^+^	0.7	54	93	WBC (counts)	0.7	55	85
**CD4**^**+**^**DR**^**+**^	**0.8**	**71**	**77**	WBC (counts)	0.7	50	93	CD3^+^CD16^+^	0.6	92	29
**CD8**^**+**^**DR**^**+**^	**0.8**	**66**	**77**	CD3^−^	0.6	96	24	CD14^+^CD64^+^(MFI)	0.6	89	36
**CD4**^**+**^	**0.8**	**54**	**100**	CD14^+^CD64^+^(MFI)	0.6	74	53	CD14^+^CD32^+^(MFI)	0.6	86	43
CD14^+^CD32^+^(MFI)	0.7	82	55	CD19^+^CD5^+^	0.6	72	59	TCRγδ^+^	0.6	86	43
WBC (counts)	0.7	79	55	CD3^+^CD16^+^	0.6	72	67	LYM	0.6	85	46
CD14^+^CD64^+^(MFI)	0.7	77	59	CD19^+^	0.6	70	59	CD4^+^CD8^+^	0.6	81	14
TCRαβ^+^	0.7	75	71	TCRαβ^+^	0.6	69	65	NEU	0.6	79	54
CD3^−^CD56^++^	0.7	66	82	CD3^−^CD16^+^CD56^−^	0.6	64	67	CD3^+^CD56^+^	0.6	69	64
CD14^+^CD16^+^/CD14^+^	0.7	57	82	CD19^+^CD23^+^	0.6	60	71	CD4^+^	0.6	67	57
CD3^+^CD16^+^	0.7	54	77	LYM	0.6	54	73	CD19^+^	0.6	67	57
NEU	0.6	97	30	CD14^+^CD16^+^DR^+^/CD14^+^CD16^+^	0.6	54	71	CD3^−^	0.6	61	71
CD14^+^CD16^+^DR^+^/CD14^+^CD16^+^	0.6	93	36	CD8^+^DR	0.6	44	77	TCRαβ^+^	0.6	46	71
LYM	0.6	89	32	CD3^−^CD16^−^CD56^+^	0.6	40	87	CD14^+^CD16^+^/CD14^+^	0.6	36	86
CD3^+^CD56^+^	0.6	85	41	CD4^+^	0.6	36	94	CD3^−^CD16^−^CD56^+^	0.5	100	15
CD3^−^CD16^+^CD56^+^	0.6	84	41	CD3^−^CD56^++^	0.6	33	100	CD8^+^	0.5	78	43
CD3^+^	0.6	63	59	NEU	0.6	24	100	CD19^+^CD23^+^	0.5	78	36
MON	0.6	62	73	CD14^+^CD16^+^/CD14^+^	0.5	96	18	CD14^+^CD16^+^DR^+^/CD14^+^CD16^+^	0.5	69	43
EOS	0.6	54	64	TCRγδ^+^	0.5	82	29	CD3^−^CD16^+^CD56^−^	0.5	63	62
CD3^−^CD16^+^ & CD3^−^CD56^+^	0.6	52	77	CD8^+^	0.5	80	41	CD4^+^DR^+^	0.5	53	71
CD19^+^CD5^−^	0.6	41	91	CD19^+^CD5^−^	0.5	80	41	CD19^+^CD5^−^	0.5	53	64
CD3^−^CD16^+^CD56^−^	0.6	39	91	CD4^+^CD8^+^	0.5	68	41	CD4^+^CD25^+^	0.5	53	71
CD19^+^CD23^+^	0.6	35	96	CD4^+^CD25^+^	0.5	60	53	EOS	0.5	39	82
CD19^+^CD5^+^	0.5	47	68	CD14^+^CD32^+^(MFI)	0.5	44	71	CD19^+^CD5^+^	0.5	33	86
CD4^+^CD8^+^	0.5	27	91	MON	0.5	44	71	CD8^+^DR	0.5	28	86
CD3^−^CD16^−^CD56^+^	0.5	26	91	CD4^+^DR^+^	0.5	40	77	MON	0.5	24	100
CD19^+^	0.5	16	100	EOS	0.5	29	93	CD3^−^CD56^+^	0.5	14	100

CTL = Uninfected infant controls (n = 22); TOXO = Infants with congenital toxoplasmosis (n = 68); NL = no retinochoroidal lesion (n = 18); L = retinochoroidal lesion (n = 50); AL = active retinochoroidal lesion (n = 14); CL = cicatricial retinochoroidal lesion (n = 36); AUC = Area under the ROC curve; Se = Sensitivity; Sp = Specificity. WBC = White Blood Cells; LYM = Lymphocytes; MON = Monocytes; NEU = Neutrophils; EOS = Eosinophils. Bold values and letters correspond to biomarkers with high accuracy (>0.8).

^a^Cut-off: CD4^+^CD25^+^ = 4.8; TCRγδ^+^ = 4.1; CD8^+^ = 23.2 ; CD4^+^DR^+^ = 5.4; CD8^+^DR^+^ = 14.1; CD4^+^ = 33.8; CD14^+^CD32^+^(MFI) = 518.3; WBC (counts) = 9,350; CD14^+^CD64^+^(MFI) = 557.5; TCRαβ^+^ = 59.5; CD3^−^CD56^++^ = 1.1; CD14^+^CD16^+^/CD14^+^ = 12.1; CD3^+^CD16^+^ = 0.9; NEU = 28; CD14^+^CD16^+^DR^+^/CD14^+^CD16^+^ = 63.7; LYM = 58.2; CD3^+^CD56^+^ = 0.4; CD3^−^CD16^+^CD56^+^ = 4.8; CD3^+^ = 66.8; MON = 10; EOS = 3.7; CD3^−^CD16^+^ & CD3^−^CD56^+^ = 18.5; CD19^+^CD5^−^ =  6.0; CD3^−^CD16^+^CD56^−^ = 8.8; CD19^+^CD23^+^ = 10.5; CD19^+^CD5^+^ = 12.7; CD4^+^CD8^+^ = 0.8; CD3^−^CD16^−^CD56^+^ = 2.3; CD19^+^ = 11.9.

^b^Cut-off: CD3^−^CD16^+^ & CD3^−^CD56^+^ = 20.4; CD3^+^CD56^+^ = 0.5; CD3^−^CD16^+^CD56^+^ = 9.7; WBC (counts) = 12,360; CD3^+^ = 75.8; CD14^+^CD64^+^(MFI) = 566.4; CD19^+^CD5^+^ = 14.2 CD3^+^CD16^+^ = 0.3; CD19^+^ = 2.6; TCRαβ^+^ = 54.4; CD3^−^CD16^+^CD56^−^ = 5.9; CD19^+^CD23^+^ = 12.9; LYM = 70.2; CD14^+^CD16^+^DR^+^/CD14^+^CD16^+^ = 80.8; CD8^+^DR^+^ = 32.6; CD3^−^CD16^−^CD56^+^ = 1.7; CD4^+^ = 28.8; CD3^−^CD56^++^ = 0.9; NEU = 10; CD14^+^CD16^+^/CD14^+^ = 26.5; TCRδγ^+^ = 11.9; CD8^+^ = 24.2; CD19^+^CD5^−^ = 4.5; CD4^+^CD8^+^ = 0.3; CD4^+^CD25^+^ = 3.9; CD14^+^CD32^+^(MFI) = 481.8; MON = 10.3; CD4^+^DR^+^ = 10.2; EOS = 2.8.

^c^Cut-off: CD3^−^CD16^+^ & CD3^−^CD56^+^ = 24.9; CD3^−^CD16^+^CD56^+^ = 7.3; WBC (counts) = 12,160; CD3^+^CD16^+^ = 0.4; CD14^+^CD64^+^(MFI) = 630.7; CD14^+^CD32^+^(MFI) = 356.6; TCRδγ^+^ = 4.4; LYM = 63.1; CD4^+^CD8^+^ = 0.8; NEU = 17; CD3^+^CD56^+^ = 0.9; CD4^+^ = 29.8; CD19^+^ = 17.6; CD3^+^ = 62.6; TCRαβ^+^ = 53.5; CD14^+^CD16^+^/CD14^+^ = 10; CD3^−^CD16^−^CD56^+^ = 4.9; CD8^+^ = 30.8; CD19^+^CD23^+^ = 17.2; CD14^+^CD16^+^DR^+^/CD14^+^CD16^+^ = 86.9; CD3^−^CD16^+^CD56^−^ = 7.2; CD4^+^DR^+^ = 7.9; CD19^+^CD5^−^ = 6.7; CD4^+^CD25^+^ = 3.6; EOS = 2.9; CD19^+^CD5^+^ = 14.2; CD8^+^DR^+^ = 10.9; MON = 13.4; CD3^−^CD56^++^ = 0.3.

### Screening of *T. gondii*-specific intracellular cytokines as complementary biomarkers for early diagnosis and prognosis of congenital toxoplasmosis

Intracellular cytokines were analyzed in monocytes, neutrophils, NK-cells, B cells and T-cells by flow cytometry upon short-term *T. gondii* antigen stimulation of whole blood samples in vitro, carried out 30–45 days after birth. The global accuracy (AUC), sensitivity (Se) and specificity (Sp) of these biomarkers to segregate “TOXO from CTL” as well as “NL from L” and “AL from CL” are provided in the Table 3. The results indicate that the *STAg*/CC index for IFN-γ^+^CD4^+^ T-cells, IL-12^+^ Monocytes and IL-5^+^ Neutrophlis displayed high accuracy (AUC > 0.8) to discriminate TOXO from CTL individuals. For early prognosis purposes, the *STAg*/CC index for IL-5^+^CD4^+^ T-cells and IL-17A^+^CD4^+^ T-cells showed high performance (AUC > 0.8) to discriminate NL from L. Additionally, the *STAg*/CC index for IFN-γ^+^ NK-cells displayed high accuracy (AUC > 0.8) to discriminate AL from CL (Table 3).

**Table 3:** Screening of *T. gondii*-specific intracellular cytokines as complementary biomarkers for early diagnosis and prognosis of congenital toxoplasmosis.

Diagnosis				Early prognosis							
Biomarkers^a^ (%)	CTL × TOXO			Biomarkers^b^ (%)	TOXO (NL × L)			Biomarkers^c^ (%)	TOXO (AL × CL)		
	AUC	Se (%)	Sp (%)		AUC	Se (%)	Sp (%)		AUC	Se (%)	Sp (%)
**IFN-γ**^**+**^**CD4**^**+**^	**0.8**	**79**	**80**	**IL-5**^**+**^**CD4**^**+**^	**0.8**	**65**	**90**	**IFN-γ**^**+**^**NK**	**0.9**	**93**	**83**
**IL-12**^**+**^**MON**	**0.8**	**78**	**70**	**IL-17A**^**+**^**CD4**^**+**^	**0.8**	**62**	**100**	IL-4^+^CD4^+^	0.7	86	50
**IL-5**^**+**^**NEU**	**0.8**	**65**	**88**	IL-4^+^CD19	0.7	100	29	IL-1β^+^MON	0.7	68	80
IL-1β^+^MON	0.7	93	56	IL-8^+^CD4^+^	0.7	81	50	IL-8^+^CD8^+^	0.7	68	71
IL-17A^+^NEU	0.7	78	56	IFN-γ^+^CD4^+^	0.7	74	60	IL-8^+^CD4^+^	0.7	67	69
IL-10^+^NEU	0.7	76	67	IL-4^+^NEU	0.7	63	78	TNF^+^CD19^+^	0.7	63	69
TNF^+^NEU	0.7	63	88	TNF^+^MON	0.7	63	78	IL-4^+^NK	0.7	60	73
TNF^+^MON	0.7	55	90	IL-5^+^CD8^+^	0.7	48	88	TNF^+^CD4^+^	0.7	52	79
IFN-γ^+^CD8^+^	0.7	45	100	IL-5^+^NEU	0.7	41	100	IL-4^+^NEU	0.7	47	85
IFN-γ^+^NK	0.7	44	100	IL-10^+^CD4^+^	0.6	98	30	IL-10^+^CD8^+^	0.6	96	29
IL-10^+^CD19^+^	0.6	90	44	TNF^+^CD8^+^	0.6	80	44	IL-4^+^CD8^+^	0.6	96	35
IL-4^+^CD19^+^	0.6	80	40	IFN-γ^+^CD8^+^	0.6	79	44	IL-6^+^NEU	0.6	75	46
IL-4^+^MON	0.6	78	50	TNF^+^CD4^+^	0.6	71	50	IL-10^+^MON	0.6	75	47
IL-10^+^MON	0.6	73	60	IL-6^+^MON	0.6	68	56	TNF^+^CD8^+^	0.6	73	59
IL-5^+^CD8^+^	0.6	71	56	IL-10^+^CD19^+^	0.6	63	71	TNF^+^NEU	0.6	71	67
IL-4^+^CD4^+^	0.6	70	60	TNF^+^NK	0.6	48	83	IL-10^+^NEU	0.6	70	63
IL-4^+^NEU	0.6	67	63	IL-1β^+^MON	0.6	47	89	IL-10^+^CD4^+^	0.6	63	63
IL-8^+^NEU	0.6	66	78	IL-17A^+^NEU	0.6	42	86	TNF^+^NK	0.6	60	67
IL-6^+^MON	0.6	65	60	IL-17A^+^CD8^+^	0.6	35	88	IL-10^+^CD19^+^	0.6	58	69
IL-1β^+^NEU	0.6	61	71	IL-1β^+^NEU	0.6	31	100	IL-4^+^MON	0.6	52	73
IL-4^+^CD8^+^	0.6	59	67	IFN-γ^+^NK	0.6	23	100	TNF^+^MON	0.6	48	79
TNF^+^CD8^+^	0.6	58	67	IL-8^+^CD8^+^	0.6	21	100	IL-17A^+^NEU	0.6	47	86
TNF^+^NK	0.6	58	100	TNF^+^NEU	0.5	100	11	IL-6^+^MON	0.6	45	79
IL-17A^+^CD4^+^	0.6	50	78	IL-4^+^CD8^+^	0.5	95	22	IFN-γ^+^CD4^+^	0.5	100	19
IL-8^+^CD4^+^	0.6	40	80	IL-10^+^MON^+^	0.5	80	44	IL-5^+^CD4^+^	0.5	96	18
TNF^+^CD4^+^	0.6	33	90	IL-4^+^CD4^+^	0.5	73	44	IL-5^+^CD8^+^	0.5	91	29
IL-17A^+^CD8^+^	0.6	33	100	IL-12^+^MON	0.5	56	67	IL-17A^+^CD8^+^	0.5	91	25
TNF^+^CD19^+^	0.6	26	100	IL-10^+^NEU	0.5	47	89	IL-12^+^MON	0.5	81	33
IL-5^+^CD4^+^	0.5	64	50	IL-4^+^MON	0.5	36	89	IL-1β^+^NEU	0.5	69	46
IL-6^+^NEU	0.5	45	75	IL-6^+^NEU	0.5	27	100	IFN-γ^+^CD8^+^	0.5	62	65
IL-8^+^CD8^+^	0.5	35	100	IL-10^+^CD8^+^	0.5	27	89	IL-17A^+^CD4^+^	0.5	55	64
IL-4^+^NK	0.5	34	100	TNF^+^CD19^+^	0.5	24	100	IL-8^+^NEU	0.5	44	80
IL-10^+^CD4^+^	0.5	18	100	IL-8^+^NEU	0.5	24	100	IL-5^+^NEU	0.5	30	88
IL-10^+^CD8^+^	0.5	16	100	IL-4^+^NK	0.5	23	100	IL-4^+^CD19^+^	0.5	24	92

CTL = Uninfected infant controls (n = 10); TOXO = Infants with congenital toxoplasmosis (n = 51); NL = no retinochoroidal lesion (n = 10); L = retinochoroidal lesion (n = 41); AL = active retinochoroidal lesion (n = 16); CL = cicatricial retinochoroidal lesion (n = 25); AUC = Area under the ROC curve; Se = Sensitivity; Sp = Specificity. NK-cells: CD16^+^FSC^Low^SSC^Low^; MON = Monocytes: CD14^+^ cells; NEU = Neutrophils SSC^High^CD16^+^ or CD14^−^ cells. Bold values and letters correspond to biomarkers with high accuracy (>0.8).

^a^Cut-off: IFN-γ^+^CD4^+^ = 1.3; IL-12^+^MON = 1.2; IL-5^+^NEU = 0.9; IL-1β^+^MON = 0.5; IL-17A^+^NEU = 0.9; IL-10^+^NEU = 0.9; TNF^+^NEU = 0.9; TNF^+^MON = 4.0; IFN-γ^+^CD8^+^ = 1.3; IFN-γ^+^NK = 1.7; IL-10^+^CD19^+^ = 0.7; IL-4^+^CD19^+^ = 0.8; IL-4^+^MON = 1.0; IL-10^+^MON = 1.3; IL-5^+^CD8^+^ = 0.9; IL-4^+^CD4^+^ = 1.1; IL-4^+^NEU = 0.9; IL-8^+^NEU = 3.4; IL-6^+^MON = 1.3; IL-1β^+^NEU = 3.8; IL-4^+^CD8^+^ = 1.0; TNF^+^CD8^+^ = 1.1; TNF^+^NK = 1.2; IL-17A^+^CD4^+^ = 1.2; IL-8^+^CD4^+^ = 1.2; TNF^+^CD4^+^ = 1.9; IL-17A^+^CD8^+^ = 0.9; TNF^+^CD19^+^ = 1.7; IL-5^+^CD4^+^ = 0.9; IL-6^+^NEU = 1.1; IL-8^+^CD8^+^ = 1.6; IL-4^+^NK = 0.8; IL-10^+^CD4^+^ = 1.7; IL-10^+^CD8^+^ = 1.5.

^b^Cut-off: IL-5^+^CD4^+^ = 1.1; IL-17A^+^CD4^+^ = 1.2; IL-4^+^CD19^+^ = 0.5; IL-8^+^CD4^+^ = 1.4; IFN-γ^+^CD4^+^ = 2.8; IL-4^+^NEU = 1.2; TNF^+^MON = 6.0; IL-5^+^CD8^+^ = 1.0; IL-5^+^NEU = 2.3; IL-10^+^CD4^+^ = 2.2; TNF^+^CD8^+^ = 1.5; IFN-γ^+^CD8^+^ = 1.9; TNF^+^CD4^+^ = 1.8; IL-6^+^MON = 3.1; IL-10^+^CD19^+^ = 1.1; TNF^+^NK = 1.1; IL-1β^+^MON = 1.2; IL-17A^+^NEU = 2.6; IL-17A^+^CD8^+^ = 1.2; IL-1β^+^NEU = 1.7; IFN-γ^+^NK = 0.9; IL-8^+^CD8^+^ = 0.8; TNF^+^NEU = 5.1; IL-4^+^CD8^+^ = 0.5; IL-10^+^MON^+^ = 1.2; IL-4^+^CD4^+^ = 1.1; IL-12^+^MON = 2.2; IL-10^+^NEU = 1.0; IL-4^+^MON = 1.6; IL-6^+^NEU = 2.4; IL-10^+^CD8^+^ = 1.2; TNF^+^CD19^+^ = 1.8; IL-8^+^NEU = 1.5; IL-4^+^NK = 0.5.

^c^Cut-off: IFN-γ^+^NK = 1.3; IL-4^+^CD4^+^ = 0.9; IL-1β^+^MON = 1.2; IL-8^+^CD8^+^ = 1.2; IL-8^+^CD4^+^ = 1.1; TNF^+^CD19^+^ = 1.1; IL-4^+^NK = 1.1; TNF^+^CD4^+^ = 1.3; IL-4^+^NEU = 0.6; IL-10^+^CD8^+^ = 0.7; IL-4^+^CD8^+^ = 1.4; IL-6^+^NEU = 1.6; IL-10^+^MON = 1.4; TNF^+^CD8^+^ = 1.2; TNF^+^NEU = 1.3; IL-10^+^NEU = 1.4; IL-10^+^CD4^+^ = 1.0; TNF^+^NK = 1.1; IL-10^+^CD19^+^ = 1.0; IL-4^+^MON = 1.2; TNF^+^MON = 6.0; IL-17A^+^NEU = 1.2; IL-6^+^MON = 1.1; IFN-γ^+^CD4^+^ = 0.8; IL-5^+^CD4^+^ = 0.5; IL-5^+^CD8^+^ = 1.7; IL-17A^+^CD8^+^ = 1.8; IL-12^+^MON = 1.3; IL-1β^+^NEU = 1.9; IFN-γ^+^CD8^+^ = 1.2; IL-17A^+^CD4^+^ = 1.3; IL-8^+^NEU = 2.1; IL-5^+^NEU = 4.2; IL-4^+^CD19^+^ = 1.8.

Aiming at screening complementary biomarkers for late prognosis of congenital toxoplasmosis, the ex vivo features of circulating leukocytes and the in vitro profile of intracellular cytokines were analyzed in whole blood samples collected 1 year after birth from TOXO to search for putative attributes with applicability as late prognosis biomarkers to distinguish “NL from L”. The results are presented in the Supplementary Table 1. All biomarkers evaluated display moderate (AUC = 0.7) or low accuracy indices (AUC < 0.7) to discriminate NL from L (Supplementary Table 1).

### Detailed performance of selected biomarkers for early complementary diagnosis of congenital toxoplasmosis

**Figure 1:**
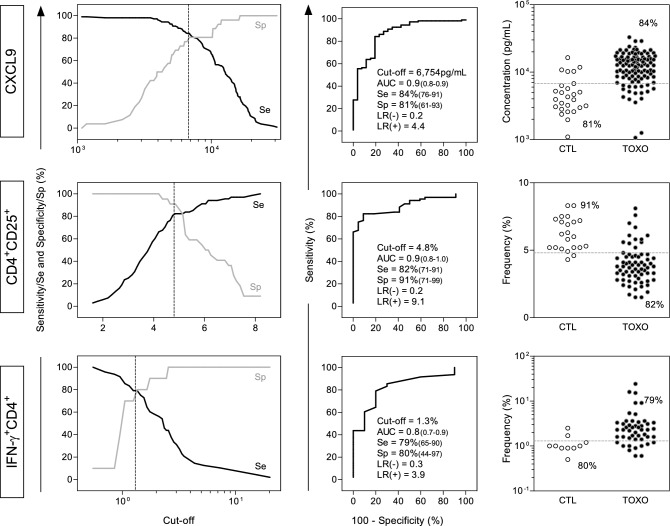
Performance of immunological biomarkers for early diagnosis of congenital toxoplasmosis. Serum levels of CXCL9 (pg/mL) and percentages of circulating CD4^+^CD25^+^ T-cells and IFN-γ^+^CD4^+^ T-cells were measured as described in “Population, material and methods”. Two-graph Receiver Operating-Characteristics (TG-ROC) were plotted based on the sensitivity (Se) and specificity (Sp) at the y axis versus cut-off at the x axis. The dotted lines indicate the cut-off with highest accuracy. Receiver Operating-Characteristics (ROC) curves were plotted considering the sensitivity (Se) and the complement of the specificity (100-Sp%) along a range of cut-offs. The area under the curve (AUC) indicates the global accuracy of each biomarker in diagnosing congenital toxoplasmosis by segregating TOXO from CTL. The performance indices (Cutt-off; Area Under the Curve—AUC; Sensitivity (Se); Specificity (Sp); Likelihood Ratio—LR(−)/LR(+) for the three selected biomarkers are provided in the figure. Scatter plots illustrate the levels of CXCL9 (pg/mL) as well as the frequency of CD4^+^CD25^+^ T-cells and IFN-γ^+^CD4^+^ T-cells in infants with congenital toxoplasmosis (TOXO, dark circles, n = 90) and age-matching healthy controls (CTL, white circles, n = 24). The dotted line represents the cut-off previously selected by TG-ROC and ROC curve analysis. The frequencies of TOXO samples (Se) and CTL (Sp) segregated by the cut-offs are displayed in each scatter plot.

Detailed performance information, including two-graph ROC curves (TG-ROC), ROC curve indices and scatter plot distribution for the biomarkers selected during screening analysis (CXCL9, CD4^+^CD25^+^ T-cells and IFN-γ^+^CD4^+^ T-cells) is presented in the Fig. 1. The TG-ROC curves were employed to define the cut-offs for each biomarker, able to segregate TOXO from CTL with the highest accuracy (Fig. 1, left panels). Data analysis indicated that CXCL9 levels above 6754 pg/ml, frequency of CD4^+^CD25^+^ T-cells higher than 4.8% and *STAg*/CC index for IFN-γ^+^CD4^+^ T-cells superior than 1.3% are the most reliable cut-offs to discriminate TOXO from CTL (Fig. 1, middle panels). The ROC curve parameters underscored the high performance for these biomarkers, (AUC = 0.9; 0.9 and 0.8, respectively) (Fig. 1, middle panels). Scatter plot distributions of individual values further illustrates the ability of CXCL9, CD4^+^CD25^+^ T-cells and IFN-γ^+^CD4^+^ T-cells to distinguish TOXO from CTL (Fig. 1, right panels).

### Performance of combined biomarkers for early diagnosis of congenital toxoplasmosis

**Figure 2:**
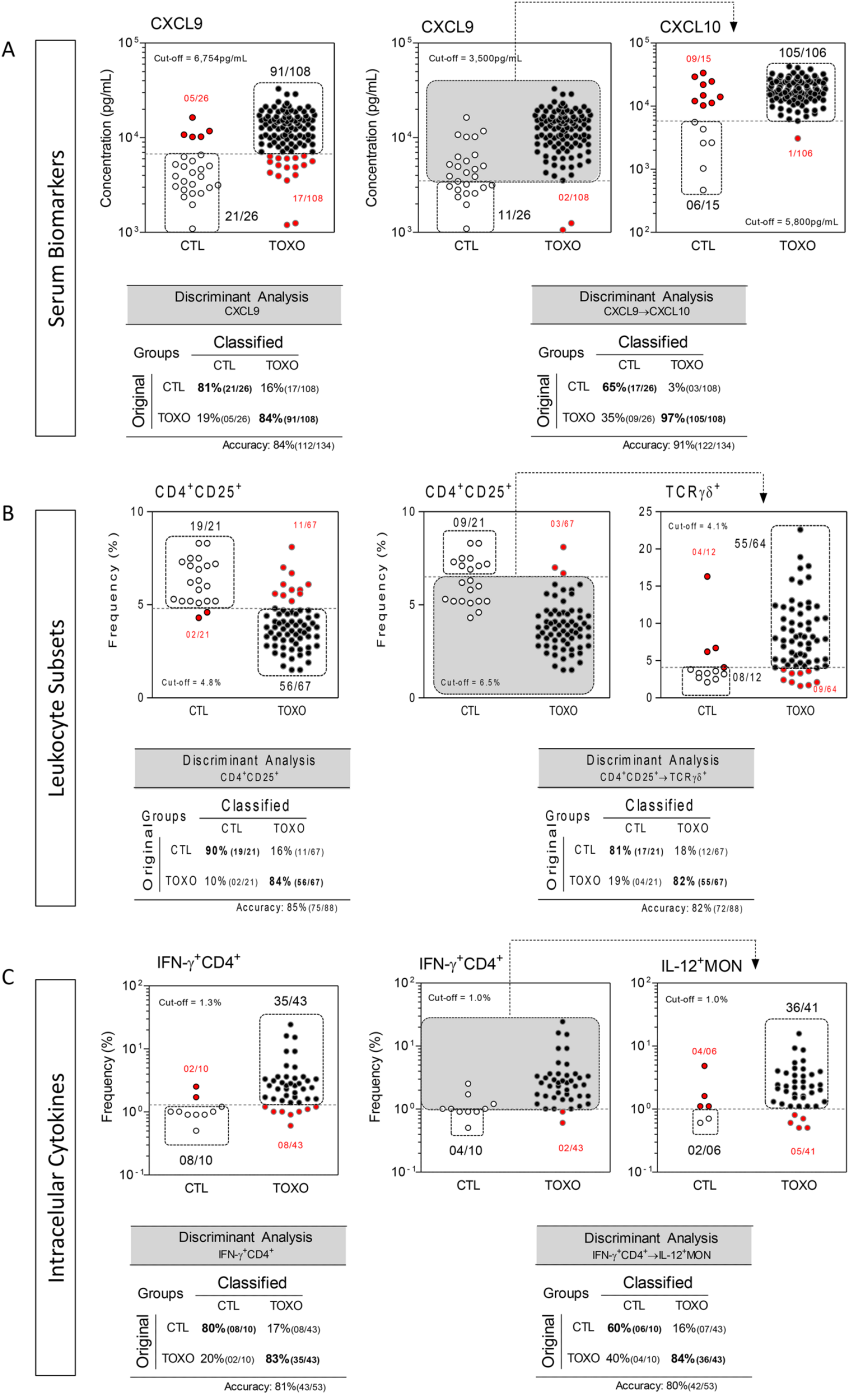
Performance of single and combined stepwise biomarker analysis for early diagnosis of congenital toxoplasmosis. Three sets of biomarkers including (**A**) serum chemokines, (**B**) leukocyte subsets and (**C**) intracellular cytokines were evaluated as single and stepwise parameters to segregate TOXO from CTL. Single biomarker analysis are displayed by individual scatter plots for serum levels of CXCL9 (pg/mL) and percentages of circulating CD4^+^CD25^+^ T-cells and IFN-γ^+^CD4^+^ T-cells in infants with congenital toxoplasmosis (TOXO, dark circles, “n” is indicated for each biomarker) and healthy controls (CTL, white circles, “n” is indicated for each biomarker). The dotted line represents the cut-offs previously selected by ROC curve analysis. The proportion of TOXO and CTL across the cut-offs are shown in each scatter plot. False-positive and false-negative results are underscored by red circles. Stepwise biomarker analysis was performed using the top two biomarkers with higher performance during single analysis. Novel cut-off edges were defined for each root attribute (CXCL9, CD4^+^CD25^+^ T-cells and IFN-γ^+^CD4^+^ T-cells) to improve the sensitivity for screening purposes. The second biomarker (CXCL10, γδ T-cells and IL-12^+^MON, respectively) was employed as a stringent attribute to provide complementary specificity. Gray backgrounds display patients selected for the second round of analysis. Data analysis was carried out with groups of infants with paired measurements (“n” is provided for each pair of biomarkers). Discriminant analysis for single and stepwise biomarker analysis are provided below each scatter plot. Accuracy of discriminant analysis is displayed on the lower left corner of each inserted table.

Attempting to improve the performance of immunological biomarkers as complementary tools for early diagnosis of congenital toxoplasmosis, the top two biomarkers of each set of parameters were combined in a sequential stepwise algorithm (Fig. 2). For this purposes, novel cut-off edges were defined for each root attribute (CXCL9, CD4^+^CD25^+^ T-cells and IFN-γ^+^CD4^+^ T-cells) to improve the sensitivity for screening purposes. A second biomarker (CXCL10, γδ T-cells and IL-12^+^MON, respectively) was then employed as a stringent attribute to provide complementary specificity. The accuracy obtained for using single or combined biomarkers was then compared by discriminant analysis. Data analysis was carried out with groups of infants with paired measurement of CXCL9:CXCL10 (TOXO = 108 and CTL = 26), CD4^+^CD25^+^ T-cells: γδ T-cells (TOXO = 67 and CTL = 21) and IFN-γ^+^CD4^+^ T-cells:IL-12^+^MON (TOXO = 43 and CTL = 10). The results demonstrated a slight increase in the overall performance with the use of combined CXCL9 → CXCL10 (Accuracy = 91%) as compared to the use of CXCL9 alone (Accuracy = 84%) (Fig. 2 A). However, decreased accuracy was obtained for the combination of the biomarkers CD4^+^CD25^+^ T-cells → γδ T-cells = 84% and IFN-γ^+^CD4^+^ T-cells → IL-12^+^MON = 84% as compared to the use of single parameters (CD4^+^CD25^+^ T-cells = 85% and IFN-γ^+^CD4^+^ T-cells = 81%) (Fig. 2B,C).

**Figure 3:**
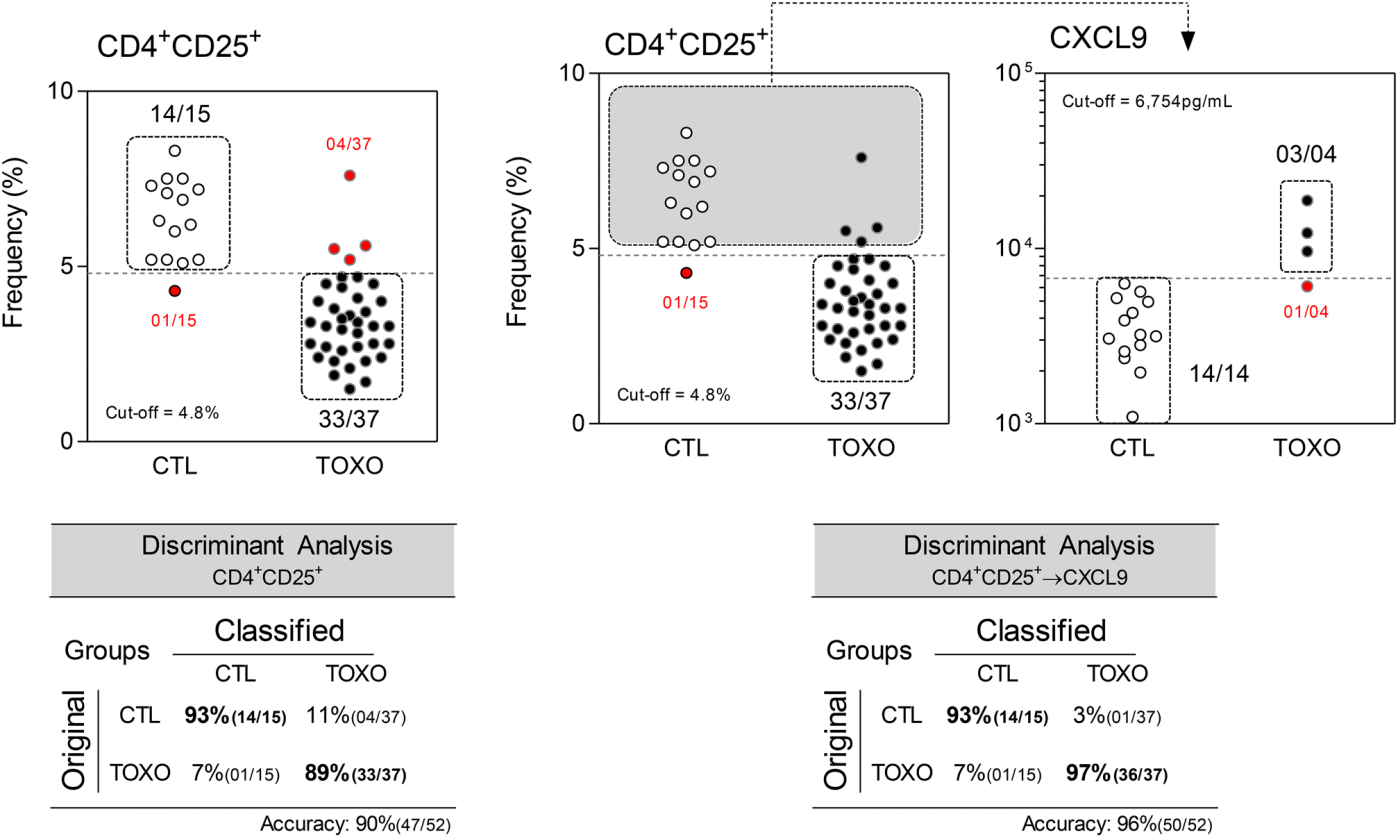
Performance of single analysis of CD4^+^CD25^+^ T-cells and combined stepwise analysis of CD4^+^CD25^+^ T-cells and CXCL9 for early diagnosis of congenital toxoplasmosis. Scatter plot illustrate the single analysis of CD4^+^CD25^+^ T-cells in infants with congenital toxoplasmosis (TOXO, dark circles, n = 37) and age-matching healthy controls (CTL, white circles, n = 15). The dotted line represents the cut-off previously selected by TG-ROC and ROC curve analysis. The frequencies of TOXO samples (Se) and CTL (Sp) segregated by the cut-offs are displayed in each scatter plot. False-positive and false-negative results are underscored by red circles. Stepwise biomarker analysis was carried out using the frequency of circulating CD4^+^CD25^+^ T-cells percentages for screening and the serum levels of CXCL9 as a second step to segregate infants with congenital toxoplasmosis (TOXO, dark circles, n = 37) from age-matched healthy controls (CTL, white circles, n = 15). The dotted line represents the cut-off previously defined by the ROC curve analysis for single biomarker use. Gray backgrounds display patients selected for the second round of analysis. The proportion of TOXO and CTL across the cut-offs are shown in each scatter plot. The dotted line rectangles show the number of cases accounted in the discriminant analysis as accurate results. The discriminant analysis results, including the single and stepwise biomarker analysis, are demonstrated in the inserted tables below each scatter plot.

Additional analysis was carried out with the stepwise combination of CD4^+^CD25^+^ T-cells → CXCL9, using a selected group of infants (TOXO = 37 and CTL = 15) with paired measurement of these attributes. The results demonstrated a relevant increase in the accuracy for CD4^+^CD25^+^ T-cells → CXCL9 combination (Accuracy = 96%) as compared with CD4^+^CD25^+^ T-cells used as a single parameter (Accuracy = 90%) (Fig. 3).

### *Performance of IFN-γ*^+^*CD4*^+^*plus IFN-γ*^+^*CD8*^+^*T-cells as early diagnosis of congenital toxoplasmosis*

**Figure 4:**
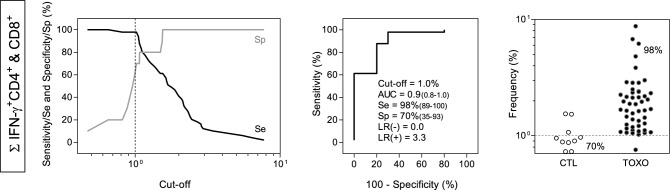
Performance of IFN-γ-producing T-cells (∑ IFN-γ^+^ CD4^+^ & CD8^+^) for early diagnosis of congenital toxoplasmosis. TG-ROC was plotted based on the at the y axis versus cut-off at the x axis. The vertical dotted line represents the cut-off with highest accuracy. ROC curves was plotted considering the sensitivity (Se%) and the complement of the specificity (100-Sp%). The performance indices (Cut-off; Area Under the Curve—AUC; Sensitivity (Se); Specificity (Sp); Likelihood Ratio—LR(−)/LR(+) are provided in the figure. Scatter plots illustrate the percentages of IFN-γ^+^ T-cells (∑ IFN-γ^+^ CD4^+^ & CD8^+^) in infants with congenital toxoplasmosis (TOXO, dark circles, n = 49) and age-matching healthy controls (CTL, white circles, n = 10). The dotted line represents the cut-off previously selected by TG-ROC and ROC curve analysis. The frequencies of TOXO samples (Se) and CTL (Sp) segregated by the cut-offs are displayed in the scatter plot.

The performance of *T. gondii*-induced IFN-γ^+^ production by T-cells observed upon short-term in vitro stimuli was further evaluated as complementary biomarker for early diagnosis of congenital toxoplasmosis. For this purpose, the sum of IFN-γ^+^CD4^+^ T-cells and IFN-γ^+^CD8^+^ T-cells (∑ IFN-γ^+^ CD4^+^ & CD8^+^) was calculated and the performance assessed by TG-ROC and ROC curve analysis (Fig. 4). The cut-off edge (*STAg*/CC Index = 1%) was defined by the TG-ROC analysis as the most reliable value to obtain the highest performance indices. A high global accuracy (AUC = 0.9) along with elevated sensitivity (Se = 98%) and moderate specificity (Sp = 70%) were obtained (Fig. 4).

### *Performance of IL5*^+^*CD4*^+^*T-cells and IFN-γ*^+^*NK-cells for early prognosis of congenital toxoplasmosis*

**Figure 5:**
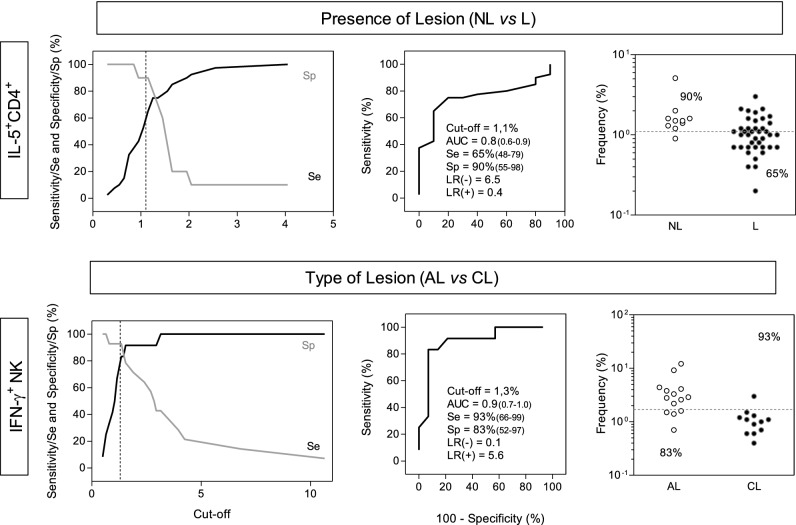
Performance of intracellular cytokines produced by T-cells for the early prognosis of ocular congenital toxoplasmosis. The selected biomarkers, IL-5^+^CD4^+^ T-cells and IFN-γ^+^NK-cells, were evaluated for their performance as novel laboratorial parameters for early prognosis of ocular congenital *T. gondii* infection. The performance of IL-5^+^CD4^+^ T-cells was tested to discriminate infants with congenital toxoplasmosis with (L) from those without (NL) retinochoroidal lesions. The frequency of IFN-γ^+^NK-cells was tested for its ability to segregate infants with active (AL) or cicatricial (CL) retinochoroidal lesions. TG-ROC was built considering the sensitivity (Se) and specificity (Sp) at the y axis versus cut-off at the x axis. The vertical dotted line shows the cut-off with highest accuracy. ROC curves were plotted considering the sensitivity (Se%) and the complement of the specificity (100-Sp%). The performance indices (Cut-off; Area Under the Curve—AUC; Sensitivity (Se); Specificity (Sp); Likelihood Ratio—LR(−)/LR(+) are provided in the figure. Scatter plots illustrate the percentages of IL-5^+^CD4^+^ T-cells in infants with (L, dark circles, n = 41) or without (NL, white circles, n = 10) retinochoroidal lesions as well as the percentage of IFN-γ^+^NK-cells in infants with active (AL, white circles, n = 14) or cicatricial retinochoroidal lesion (CL, dark circles, n = 12). The dotted line displays the cut-offs selected by TG-ROC and ROC curve analysis. The frequencies of infants above and below the cut-offs are displayed in each scatter plot.

In order to verify the operating characteristic of novel biomarkers for early prognosis of ocular congenital *T. gondii* infection, a detailed analysis of IL5^+^CD4^+^ T-cells and IFN-γ^+^NK-cells were carried out in TOXO subgroups by comparing NL vs. L and AL vs. CL, respectively (Fig. 5). The TG-ROC curves were used to determine the most appropriated *STAg*/CC Index cut-offs (IL5^+^CD4^+^ T-cells = 1.1% and IFN-γ^+^NK-cells = 1.3%) for accurate early prognosis of congenital *T. gondii* infection (Fig. 5, left panels). ROC curve analysis indicated the high performance of these biomarkers to distinguish NL from L (AUC = 0.8) and AL from CL (AUC = 0.9) (Fig. 5, middle panels). Scatter plots distribution of individual values further illustrate the ability of IL5^+^CD4^+^ T-cells and IFN-γ^+^NK-cells to accordingly categorize TOXO infants based on their status of retinochoroidal lesions (Fig. 5, right panels).

## Discussion

Congenital infection with *T. gondii* can lead to severe neurological and ophthalmological sequelae and therefore the early diagnosis is relevant to support prompt clinical management and therapeutic intervention^15^. The most common methods employed for the diagnosis of congenital toxoplasmosis in newborns and infants are serological-based detection of *T. gondii* specific IgM, IgG and IgA antibodies^18,19^. Although these classical methods may provide differential diagnosis of acute, chronic or reactivated acquired toxoplasmosis, they have little relevance for early diagnosis of congenital toxoplasmosis, specially due to passive transfer of long-term-persistence of maternal *T. gondii* specific IgG to infants at risk for congenital toxoplasmosis or contamination with some maternal anti-*T. gondii* IgM and IgA during the first days of life of the newborn^20,21^. Furthermore, the sensitivities for IgM and IgA detection do not exceed 70% and 65% of infected babies, respectively^2^.In general, a definitive serological diagnosis of congenital toxoplasmosis requires subsequent follow-up for at least one year^21,22^.

Several analytical methods have been proposed as complementary laboratory biomarkers for early diagnosis of congenital toxoplasmosis with high sensitivity and specificity aiming to improve clinical decision-making^15,17^. In the present work we have evaluated the sensitivity and specificity of a broad spectrum of immunological parameters and characterized their accuracy as complementary biomarkers for early diagnosis and prognosis of congenital toxoplasmosis. The analysis included the measurement of serum chemokines and cytokines levels along with the quantification of circulating leucocytes subsets and analysis of intracellular cytokines upon antigen-specific short-term in vitro stimulation.

Our data demonstrated that CXCL9 and CXCL10 have good performance to discriminate *T. gondii*-infected infants from age matched non-infected controls. Moreover, we have also observed that the combined analysis of CXCL9 and CXCL10 improves the accuracy of these biomarkers to diagnose toxoplasmosis. We have previously shown increased levels of serum CXCL9 and CXCL10 in *T. gondii*-infected infants early after birth^14^. High levels of chemokines have been also described in the aqueous humor from patients with primary or recurrent ocular toxoplasmosis as compared to disease-free controls^23^. It is well known that the immune response induced by *T. gondii* infection is mediated by a rich microenvironment of activated cells and soluble inflammatory mediators including chemokines, cytokines and cell factors^14,24–30^. It has been already reported that the expression of CXCL9 and CXCL10 in the retina was significantly upregulated in experimental model of ocular toxoplasmosis^28^. CXCL9 has been shown to be crucial for recruiting and activate T-cells to control *T. gondii* infection^30^. Importantly, CXCL10 increased transmigration of human monocyte-derived dendritic cell preparations infected with *T. gondii* towards human retinal endothelium^29^. These findings support the relevance of measuring CXCL9 and CXCL10 as complementary tools for the early diagnosis of congenital toxoplasmosis.

Besides the analysis of serum biomarkers, our results showed that the ex vivo analysis of circulating leucocytes subsets can be employed as complementary immunological test for early diagnosis of congenital toxoplasmosis. The assessment of γδ T-cells and CD4^+^CD25^+^ T-cells displayed high performance to distinguish TOXO from CTL. Although the combined analysis of CD4^+^CD25^+^ and γδ T-cells does not improve the accuracy of these biomarkers for early diagnosis, the evaluation of CD4^+^CD25^+^ T-cells followed of CXCL9 lead to an increase in the diagnosis performance. Previous studies have proposed the measurement of CD25^+^ T-cells upon *T. gondii*-specific stimulation as a simple, sensitive and specific test for diagnosis of congenital toxoplasmosis for cases in which the serological tests were inaccurate^8,9^. Another study showed that the evaluation of the specific T cell response, such as CD25 and HLA-DR expression, IFN-γ production by T-cells and T-cell proliferation supported the diagnosis of *T. gondii*-infected neonates, 3 months or older^9^. Regarding the activation marker CD25, our data showed rather a decrease in the frequency of circulating CD25^+^CD4^+^ T-cells from TOXO *T. gondii*-infected 30–45 days old newborns as compared to age-matched uninfected controls.

A prominent pro-inflammatory response of CD4^+^ and CD8^+^ T-cells, characterized by high levels of IFN-γ has been reported during congenital toxoplasmosis; in cases without ocular involvement as well as in those with active or cicatricial retinochoroidal lesion^12^. In this context, our results have shown that the production of IFN-γ by CD4^+^ T-cells after short-term stimulation with *T. gondii* antigen has high accuracy for the early diagnosis of congenital toxoplasmosis. Moreover, the production of IFN-γ by both CD4^+^ and CD8^+^ T-cells reveal an even higher performance for diagnosis. The analysis of secreted IFN-γ produced upon stimulation with *T. gondii* antigens, known as IGRA, has been employed as a diagnosis method of toxoplasmosis. In general, these T-cell-based tests have been considered a sensitive and specific diagnostic tool for congenital toxoplasmosis, complementary to serological tests^5, 11,17^.

In this regard, we showed that the production of IFN-γ by NK cells display high accuracy to discriminate infants with active or cicatricial retinochoroidal lesions. IFN-γ produced by NK cells is important for regulating inflammatory cell dynamics and also in driving cell differentiation for the initiation of the immune response to *T. gondii*^24^. However, in what extant this cytokine is associated with ocular involvement is still not known. Our results also showed that the production of IL-5 by CD4^+^ T-cells was able to distinguish with high accuracy infants with retinochoroidal lesions from those without ocular disease. Thus, while the assessment of IFN-γ producing NK cells provide a putative biomarker for early prognosis of congenital toxoplasmosis, the analysis of IL-5^+^CD4^+^ T-cells upon *T. gondii* antigens stimulation is a potential prognostic marker of ocular involvement in infant with congenital toxoplasmosis. Upregulation of IL-5 has been related to severe clinical features of ocular toxoplasmosis with increased levels of IL-5 found in the aqueous humor samples of patients with acute and recurrent ocular toxoplasmosis^31^.

Taken together, our findings reinforce the importance of evaluating elements of the immune response as biomarkers to define an early diagnosis and prognosis of congenital toxoplasmosis. This study screened a range of immunological assays to measure ex vivo and post stimulation *T. gondii* specific parameters defining biomarkers with high accuracy for the diagnosis and prognosis of congenital toxoplasmosis. We propose the levels of CXCL9 and CXCL10, the frequencies of CD4^+^CD25^+^ T-cells and the frequency of *T. gondii*-specific IFN-γ producing CD4^+^ T-cells presented as biomarkers able to distinguish with high accuracy infants with congenital toxoplasmosis from uninfected healthy controls. Combined analyses of CD4^+^CD25^+^ T-cells and CXCL9, and IFN-γ production by CD4^+^ and CD8^+^ T-cells have even higher accuracy as biomarkers of congenital toxoplasmosis. As for early prognosis *T. gondii*-specific IL5^+^CD4^+^ T-cells and IFN-γ produced by NK-cells displayed high accuracy to define respectively ocular involvement and acute/chronic phase of ocular toxoplasmosis in infants with congenital disease. Together, these findings support the relevance of employing the elements of the cell-mediated immune response as biomarkers with potential to endorse early diagnosis and prognosis of congenital toxoplasmosis. The analyses of these biomarkers have potential used as complementary approaches to contribute for better clinical management and therapeutic intervention during congenital toxoplasmosis. The present study may have some limitations regarding the number of samples evaluated and requires further validation in future investigation. Overall, these findings support the relevance of employing the elements of the cell-mediated immune response as biomarkers with potential to endorse early diagnosis and prognosis of congenital toxoplasmosis.

## Population, material and methods

### Study population

This study was part of a prospective investigation on neonatal screening for congenital toxoplasmosis conducted by a multidisciplinary research group (UFMG Congenital Toxoplasmosis Brazilian Group).

Air-dried whole blood samples (heel puncture) were collected immediately after birth from 146,307 newborns and used for the initial screening of anti-*T. gondii* IgM by Enzyme-Linked Immunosorbent Assay (ELISA) (Q-Preven TOXO, Symbiosis, Leme, Brazil). Individuals with positive or indeterminate anti-*T. gondii* IgM results were further selected in a non-probabilistic convenience sampling for additional peripheral blood collection. Whole blood samples were obtained at 30–45 days and one year after birth to identify putative biomarkers for early diagnosis/prognosis or late prognosis purposes, respectively. A total of 215 infants were selected, including: 177 infants with confirmed diagnosis of congenital toxoplasmosis (TOXO) and 38 infants included as a control group (CTL), with negative results for anti-*T. gondii* IgG ELFA test (ELFAVIDAS TOXO, Biomerieux, France), carried out in additional blood samples collected 12 months after birth.

Fundoscopic analysis was carried out by one of us (DVVS) at 30–45 days after birth to evaluate ophthalmological impairment as previously described^32^. Based on the ophthalmological findings, TOXO group was first categorized into two subgroups, referred as: patients with no retinochoroidal lesion (NL) and patients with retinochoroidal lesion (L). Further classification of TOXO patients was performed based on the presence of active (AL) or cicatricial retinochoroidal lesions (CL). CTL children did not present any ophthalmological impairment.

### Ethics statement

The protocols conducted in this study were approved by the local Ethics Committee (Federal University of Minas Gerais, protocol 298/06). All experiments were performed in accordance with relevant guidelines and regulations. Written informed consent was given by all mothers of infants included in this study.

### Quantification of serum chemokines and cytokines

A total of 134 samples collected at 30–45 days after birth were assayed for quantification of serum chemokines and cytokines (108 TOXO and 26 CTL) by Cytometric Bead Array according to the manufacturer’s instructions (CBA, BD Biosciences, San Jose, CA, USA). The chemokines (CXCL8, CCL2, CCL5, CXCL9 and CXCL10) were measured by the conventional CBA system and the cytokines levels (IL-1β, IL-6, TNF, IL-12, IFN-γ, IL-4, IL-5, IL-10 and IL-17A) determined using the enhanced sensitivity CBA Flex array. Sample acquisition was performed using the BD FACSVerse flow cytometer (Becton Dickinson, La Jolla, CA, USA) and data analysis carried out employing the FCAP Array Software V3.0 (Becton Dickinson, La Jolla, CA, USA). As previously described^14^, the results were expressed as pg/mL (chemokines) or fg/mL (cytokines), as assessed by the standard curve using the forth-logistic regression parameter. The limits of detection were CXCL8: 2.5, CCL2: 0.2, CCL5:1.0, CXCL9: 2.7, CXCL10: 2.8 (expressed as pg/mL), IL-1β: 274.35, IL-6: 409.62, TNF: 144.62 , IL-12: 191.48, IFN-γ: 172.09, IL-4: 238.34, IL-5: 407.19, IL-10: 152.70 and IL-17A = 239.95 (expressed as fg/mL). All patient samples were assayed in the same batch using the same standard curve to avoid inter-assay variability^14^.

### Immunophenotyping of circulating leukocytes

Peripheral blood from infants with congenital toxoplasmosis (TOXO) and noninfected infants (CTL) was collected in Heparin/EDTA tubes. A total of 90 samples (68 TOXO and 22 CTL), collected at 30–45 days after birth and 81 samples of TOXO collected one year after birth were used for immunophenotypic analysis of circulating leukocytes. Samples were processed, and leukocytes were used for ex vivo protocols, as previously described^10^. Monoclonal antibodies were used for labeling cell surface molecules: anti-CD14 (TüK4), anti-CD16 (3G8), anti-CD32 (FLI8.26) and anti-CD64 (10.1) for monocytes, anti-CD16 (3G8) and anti-CD56 (B159) for NK-cells and NKT cell subsets; anti-TCRαβ (WT31) and anti-TCRγδ (11F2), anti-CD3 (UCHT1), anti-CD4 (RPA-T4), anti-CD8 (B9.11) for T-cell subsets and anti-HLA-DR (TÜ36) as activation marker for T-cells along with anti-CD5 (L17F12), anti-CD19 (4G7) and anti-CD23 (M-L233) for B-cell subpopulations. Antibodies conjugated with fluorescein isothiocyanate (FITC), phycoerythrin (PE), or Tricolor (TC) were purchased from Invitrogen Life Technologies (Carlsbad, CA, USA) and BD Bioscience (San Diego, CA, USA). Data acquisition was performed using FACSCalibur and the results expressed as % of gated cells or mean fluorescence intensity (MFI) of cell surface marker expression. FlowJo (version 9.4.1; TreeStar, Ashland, Oregon) was used for data analysis.

### Short-term whole blood culture in vitro and analysis of intracytoplasmic cytokines

Heparinized whole blood samples (3 mL) from 61 infants (51 TOXO and 10 CTL) collected at 30–45 days after birth and 50 samples of TOXO collected one year after birth were used for in vitro short-term culture as previously described^12^. Blood samples were dispensed into polypropylene tubes and cultured with Roswell Park Memorial Institute medium (RPMI) (control culture, CC) or soluble *T. gondii* antigen (*STAg*), produced as previously described^33^, at a concentration of 5 µg/mL (*T. gondii*–stimulated culture). Samples were incubated for 12 h in a 5% CO_2_ incubator at 37 °C. Brefeldin A (Sigma, St Louis, Missouri) was added to each culture tube at a final concentration of 10 µg/mL for additional 4 h. Before immunostaining, CC and *STAg*–stimulated cells were treated with EDTA and kept at room temperature for 15 min prior intracellular cytokine analysis. After stimulation, cells were stained with the following surface antibodies: anti-CD14-TC, anti-CD16-TC, anti-CD4-TC, anti-CD8-TC or anti-CD19-TC (BD Bioscience, San Diego, CA, USA), at room temperature for 30 min. Red blood cells were then lysed, and the leukocytes were fixed with lysing/fixing solution for 10 min at room temperature. Washes were performed using phosphate-buffered saline (PBS) supplemented with 0.5% bovine serum albumin and permeabilization using PBS-saponin (PBS, 0.5% bovine serum albumin, 0.5% saponin). Fixed permeabilized cells were stained with anti-IL-8 (AS14), anti-IL-1β (AS10), anti-IL-6 (AS12), anti-TNF (Mab11), anti-IL-12 (C11.5), anti-IFN-γ (4S.B3), anti-IL-4 (8D4-8), anti-IL-5 (TRFK5), anti-IL-10 (JES3-19F1) or anti-IL-17A (SCPL1362) monoclonal antibodies conjugated with PE, for 30 min at room temperature. Cells were washed and fixed with FACS fixing solution (10 g/L paraformaldehyde, 10.2 g/L sodium cacodylate, and 6.63 g/L sodium chloride; pH 7.2). Samples were stored in the absence of light, and acquisition performed in 24 h using FACSCalibur (BD Biosciences). Each analysis was performed using at least 20,000 gated events .Results were analyzed using the FlowJo software. Cytokine secretion by different cell subsets was defined by the gating strategy: selection of lymphocytes based on their size and granularity laser scattering properties. Posteriorly, were calculated the frequency of cytokines-producing cells in lymphocytes subsets or monocytes in the nonstimulated and *STAg* cultures. Further analysis was performed to estimate the *T. gondii*–specific cytokine production as the index of *STAg*–stimulated culture divided by the CC (hereafter, the *STAg*/CC index)^12^.

### Statistical analysis

The statistical tool used to determine the cut-off point as well as the relative sensitivity and specificity indexes and the respective confidence intervals at 95% of the tests was the receiver operating characteristic (ROC curve) and Two-graph-receiver operating characteristic (TG-ROC). The definition of cut-off points for each biomarker was determined by ROC curve analysis, considering the highest possible values of sensitivity and specificity. The global accuracy was estimated considering the area under the ROC curve (AUC) categorized as low (AUC < 0.7), moderate (0.7 < AUC < 0.8) or high (AUC > 0.8). Combined analysis of biomarkers, including serum chemokines and cytokines; immunophenotypic profile of circulating leukocyte as well as intracytoplasmic cytokine patterns, were also carried out, using a sequential algorithm proposed for those attributes with higher accuracy at screening. GraphPad Prism 5.0 was used to construct the ROC and TG-ROC curves.

## Supplementary information

Supplementary Information.
