# Supplementary material for: Putative biomarkers for early diagnosis and prognosis of congenital ocular toxoplasmosis
Source: Sci Rep. 2020 Oct 7;10:16757. doi: 10.1038/s41598-020-73265-z (PMC7541609; doi:10.1038/s41598-020-73265-z)
Supplement: Supplementary file 1 — Supplementary Information. [file 41598_2020_73265_MOESM1_ESM.pdf]

Putative biomarkers for early diagnosis and prognosis of congenital ocular toxoplasmosis

Thádia Evelyn de Araújo[a,b,*], Luara Isabela dos Santos[b,c], Angelica Oliveira Gomes[d], Ana Carolina Aguiar Vasconcelos Carneiro[e], Anderson Silva Machado[e], Jordana Grazziela Coelho-dos-Reis[f], Vanessa Peruhype-Magalhães[b], Samantha Ribeiro Béla[b], Gláucia Manzan Queiroz Andrade[g,h], Daniel Vitor Vasconcelos-Santos[h,i], José Nélio Januário[h], Andréa Teixeira-Carvalho[b], Ricardo Wagner Almeida Vitor[e], Lis Ribeiro do Valle Antonelli[b,*], Eloisa Amália Vieira Ferro[a], Olindo Assis Martins-Filho[a,b], on behalf of the UFMG Congenital Toxoplasmosis Brazilian Group, - UFMG-CTBG

[a]Instituto de Ciências Biomédicas, Universidade Federal de Uberlândia, Avenida João Naves de Ávila 2121, Santa Mônica, 38408-100, Uberlândia, MG, Brazil.

[b]Instituto René Rachou, Fundação Oswaldo Cruz, Avenida Augusto de Lima, 1715, Barro Preto, 30190-002, Belo Horizonte, MG, Brazil;

[c]Faculdade de Ciências Médicas de Minas Gerais, Alameda Ezequiel Dias, 275, Centro, 30130-110, Belo Horizonte, MG, Brazil.

[d] Instituto de Ciências Biológicas e Naturais, Universidade Federal do Triângulo Mineiro, Rua Frei Paulino, 30, Nossa Sra. da Abadia, 38025-180, Uberaba, MG, Brazil.

[e]Departamento de Parasitologia, Universidade Federal de Minas Gerais, Avenida Presidente Antônio Carlos, 6627, Pampulha, 31270-901 Belo Horizonte, MG, Brazil;

[f]Departamento de Microbiologia, Instituto de Ciências Biológicas, Universidade Federal de Minas Gerais, Avenida Antônio Carlos 6627, Pampulha, 31270-901, Belo Horizonte, MG, Brazil.

[g]Departamento de Pediatria, Universidade Federal de Minas Gerais, Avenida Professor Alfredo Balena 190, Santa Efigênia, 30130-100 Belo Horizonte, MG, Brazil;

[h]Núcleo de Ações e Pesquisa em Apoio Diagnóstico (NUPAD), Universidade Federal de Minas Gerais, Avenida Professor Alfredo Balena 190, Santa Efigênia, 30130-100 Belo Horizonte, MG, Brazil.

[i]Departamento de Oftalmologia e Otorrinolaringologia, Faculdade de Medicina da UFMG, Belo Horizonte, MG, Brazil;

* Corresponding Author: Thádia Evelyn de Araújo and Lis Ribeiro do Valle Antonelli. Instituto René Rachou, Fundação Oswaldo Cruz. Avenida Augusto de Lima, 1715 – Barro Preto – Belo Horizonte – Minas Gerais – 30190-002 – Brazil – Phone: +(55) 31 33497764 Fax: +(55) 31 32953115 e-mail: thadia_evelyn@hotmail.com; lis.antonelli@fiocruz.br

Supplementary Table 1. Screening of ex vivo circulating leukocyte subsets and T. gondii-specific intracellular cytokines as complementary biomarkers for prognosis of congenital toxoplasmosis one year after birth*

CLINICAL STATUS (one year after birth)							
Leukocyte subsets$^{\#}$ (% or MFI, Counts)	TOXO (NL x L)			*T. gondii*-specific cytokines§ (%)	TOXO (NL x L)		
	AUC	Se (%)	Sp (%)		AUC	Se (%)	Sp (%)
CD14^{+}CD16^{+}DR^{+}/CD14^{+}CD16^{+}	0.7	52	82	IL-4^{+}NK	0.7	95	50
CD19^{+}CD5^{-}	0.6	89	39	IL-6^{+}MON	0.7	93	50
LYM	0.6	87	39	TNF^{+}NK	0.7	88	56
CD3^{+}	0.6	84	44	IL-4^{+}MON	0.7	82	75
CD8^{+}DR^{+}	0.6	80	47	IL-1β^{+}MON	0.7	78	75
CD4^{+}CD8^{+}	0.6	73	56	IFN-γ^{+}CD4^{+}	0.7	77	67
NEU	0.6	68	56	IFN-γ^{+}NK	0.7	76	67
CD14^{+}CD64^{+}(MFI)	0.6	68	53	IL-10^{+}CD4^{+}	0.7	68	83
CD19^{+}	0.6	65	61	IL-17A^{+}NEU	0.7	53	68
CD3^{-}CD16^{-}CD56^{+}	0.6	64	61	IL-10^{+}MON	0.6	97	33
MON	0.6	61	59	IL-17A^{+}CD8^{+}	0.6	90	33
CD3^{+}CD56^{+}	0.6	60	67	IL-17A^{+}CD4^{+}	0.6	86	50
TCR$\alpha\beta^{+}$	0.6	57	65	IL-5^{+}CD8^{+}	0.6	72	63
CD19^{+}CD23^{+}	0.6	55	67	TNF^{+}CD4^{+}	0.6	65	67
CD14^{+}CD16^{+}/CD14^{+}	0.6	49	71	TNF^{+}NEU	0.6	57	67
CD4^{+}DR^{+}	0.6	45	78	IL-8^{+}CD4^{+}	0.6	45	83
CD3^{-}CD16^{+} & CD3^{-}CD56^{+}	0.6	35	89	IL-8^{+}NEU	0.6	39	89
EOS	0.6	20	100	TNF^{+}CD8^{+}	0.6	38	89
TCR$\gamma\delta^{+}$	0.5	85	41	IL-5^{+}CD4^{+}	0.6	36	83
CD4^{+}CD25^{+}	0.5	70	44	IFN-γ^{+}CD8^{+}	0.6	34	100
CD3^{-}CD56^{++}	0.5	69	53	TNF^{+}CD19^{+}	0.6	29	100
WBC (Counts)	0.5	68	44	IL-10^{+}NEU	0.6	28	100
CD19^{+}CD5^{+}	0.5	48	72	IL-10^{+}CD8^{+}	0.6	26	100
CD8^{+}	0.5	45	72	IL-1β^{+}NEU	0.6	22	100
CD4^{+}	0.5	41	83	IL-6^{+}NEU	0.5	93	33
CD3^{+}CD16^{+}	0.5	40	78	IL-4^{+}NEU	0.5	77	44
CD3^{-}CD16^{+}CD56^{+}	0.5	40	78	IL-8^{+}CD8^{+}	0.5	74	38
CD3^{-}CD16^{+}CD56^{-}	0.5	35	89	IL-10^{+}CD19^{+}	0.5	69	50
CD14^{+}CD32^{+}(MFI)	0.5	24	94	TNF^{+}MON	0.5	66	60
-	-	-	-	IL-4^{+}CD4^{+}	0.5	53	67
-	-	-	-	IL-5^{+}NEU	0.5	51	67
-	-	-	-	IL-12^{+}MON	0.5	41	80
-	-	-	-	IL-4^{+}CD8^{+}	0.5	33	86
-	-	-	-	IL-4^{+}CD19^{+}	0.5	27	100

* Ophthalmologic findings employed to define the clincal status (NL and L) as well as immunological features were measured one year after birth. TOXO=Infants with congenital toxoplasmosis (n$^{\#}$=81; n§=50); NL=no retinochoroidal lesion (n$^{\#}$=18; n§=9); L=retinochoroidal lesion (n$^{\#}$=63; n§=41); AUC=Area under the ROC curve; Se=Sensitivity; Sp=Specificity. #Cut-off: CD14^{+}CD16^{+}DR^{+}/CD14^{+}CD16^{+}=91.7; CD19^{+}CD5^{-}=15.0; LYM=71.3; CD3^{+}=60.8; CD8^{+}DR^{+}=2.0; CD4^{+}CD8^{+}=1.1; NEU=24.0; CD14^{+}CD64^{+}(MFI)=181; CD19^{+}=21.8; CD3^{-}CD16^{-}CD56^{+}=1.3; MON=5.9; CD3^{+}CD56^{+}=1.3; TCR$\alpha\beta^{+}$=59.1; CD19^{+}CD23^{+}=11.0; CD14^{+}CD16^{+}/CD14^{+}=7.1; CD4^{+}DR^{+}=3.2; CD3^{-}CD16^{+} & CD3^{-}CD56^{+}=8.9; EOS=8.0; TCR$\gamma\delta^{+}$=8.3; CD4^{+}CD25^{+}=6.1; CD3^{-}CD56^{++}=1.6; WBC(Counts)=; CD19^{+}CD5^{+}=9.3; CD8^{+}=31.9; CD4^{+}=40.4; CD3^{+}CD16^{+}=1.9; CD3^{-}CD16^{+}CD56^{+}=5.7; CD3^{-}CD16^{+}CD56^{-}=1.6; CD14^{+}CD32^{+}(MFI)=229. §Cut-off: IL-4^{+}NK=2.3; IL-6^{+}MON=2.6;TNF^{+}NK=1.7; IL-4^{+}MON=1.8; IL-1β^{+}MON=3.5; IFN-γ^{+}CD4^{+} =1.2; IFN-γ^{+}NK=1.7; IL-10^{+}CD4^{+}=0.9; IL-17A^{+}NEU=0.9; IL-10^{+}MON=2.6; IL-17A^{+}CD8^{+}=2.5; IL-17A^{+}CD4^{+}=1.7; IL-5^{+}CD8^{+}=1.4; TNF^{+}CD4^{+}=1.1; TNF^{+}NEU=1.7; IL-8^{+}CD4^{+}=1.0; IL-8^{+}NEU=1.3; TNF^{+}CD8^{+}=1.5; IL-5^{+}CD4^{+}=0.8; IFN-γ^{+}CD8^{+}=0.7; TNF^{+}CD19^{+}=1.5; IL-10^{+}NEU=1.5; IL-10^{+}CD8^{+}=1.5; IL-1β^{+}NEU=1.0; IL-6^{+}NEU=3.0; IL-4^{+}NEU=1.7; IL-8^{+}CD8^{+}=1.4; IL-10^{+}CD19^{+}=1.3; TNF^{+}MON=2.5; IL-4^{+}CD4^{+}=0.9; IL-5^{+}NEU=1.4; IL-12^{+}MON=1.7; IL-4^{+}CD8^{+}=1.2; IL-4^{+}CD19^{+}=1.4.
